# Psychological treatments for return to work in individuals on sickness absence due to common mental disorders or musculoskeletal disorders: a systematic review and meta-analysis of randomized-controlled trials

**DOI:** 10.1007/s00420-018-1380-x

**Published:** 2018-11-24

**Authors:** Anna Finnes, Pia Enebrink, Ata Ghaderi, JoAnne Dahl, Anna Nager, Lars-Göran Öst

**Affiliations:** 1 Division of Psychology, Department of Clinical Neuroscience, Karolinska Institutet, Nobels väg 9, 171 77 Stockholm, Sweden; 2 Department of Psychology, Uppsala University, Box 1225, 751 42 Uppsala, Sweden; 3 Division of Family Medicine and Primary Health Care, Department of Neurobiology, Care Sciences and Society, Karolinska Institutet, Riddarstigen 30, Täby, 183 30 Stockholm, Sweden; 4 Department of Psychology, Stockholm University, 106 91 Stockholm, Sweden

**Keywords:** Return to work, Psychological treatment, Common mental disorders, Musculoskeletal disorders, Meta-analysis

## Abstract

**Purpose:**

Common mental disorders (CMDs) and musculoskeletal disorders are highly prevalent in the population and cause significant distress and disability, and high costs to society. The main objective of this systematic review and meta-analysis was to examine the outcome and comparative effectiveness of psychological interventions in reducing sickness absence (SA) due to CMDs or musculoskeletal disorders, compared to a waitlist control group, usual care or another clinical intervention.

**Methods:**

We reviewed 3515 abstracts of randomized controlled trials published from 1998 to 2017. Of these, 30 studies were included in the analysis.

**Results:**

The psychological interventions were overall more effective than treatment as usual in reducing SA (small effect sizes), but not compared to other clinical interventions. Results were similar for studies on CMDs and musculoskeletal pain. A few significant moderating effects were found for treatment-specific variables. However, these were  difficult to interpret as they pointed in different directions.

**Conclusion:**

There was a small but significant effect of psychological treatments in reducing SA. We identified areas of improvement such as methodological problems among the included studies and failure to specifically address RTW in the interventions that were evaluated. Clinical implications of the findings, and ways of improving methodological rigour of future studies are discussed.

**Electronic supplementary material:**

The online version of this article (10.1007/s00420-018-1380-x) contains supplementary material, which is available to authorized users.

## Introduction

Common mental disorders (CMDs) and musculoskeletal disorders are highly prevalent health problems causing significant distress and disability (Vos et al. 2012), and high costs to society. Public spending on total sickness absence (SA) benefit totals 2% of the gross domestic product on average across the OECD countries, and as high as 4–5% in Norway, the Netherlands, and Sweden (OECD 2010). The majority of SA days is due to musculoskeletal disorders and CMDs such as depression and anxiety (OECD 2008). The prevalence of CMDs varies across countries due to definition and assessment methods but the World Health Organization (WHO) recently estimated the prevalence of depression to 4.4% and of anxiety disorders to 3.6% for the global population (WHO 2017). Depression is currently ranked by WHO (2017) as the single largest contributor to global disability and the number of people suffering from depression and anxiety is rising. For instance, the increase in new SA spells due to CMDs in Sweden was 59% between the years 2010 and 2015. During the same period, the increase in musculoskeletal disorders was 18% (Swedish Social Insurance Agency 2016). Musculoskeletal disorders are the second most common cause of disability worldwide, with low back and neck pain being the most frequent conditions (Vos et al. 2012). In summary, musculoskeletal disorders and CMDs accounts for most of the SA spells. The increasing CMD prevalence rates and disability costs indicate a need for policy-makers to advance disability policy. Considerable economic savings may be achieved both from an individual and societal perspective by increasing our knowledge about how to assist individuals on SA with improved mental health and returning to work.

Today, psychological treatments, such as cognitive behaviour therapy (CBT), interpersonal therapy (IPT), and psychodynamic therapy (PDT), are applied to a wide range of psychological, somatic and behavioural problems. There is strong support for the effectiveness of CBT when targeting various CMDs including mood and anxiety disorders (Butler et al. 2006). For musculoskeletal disorders, the predominant contemporary model consists of an integrative and multidimensional biopsychosocial theoretical framework (Gatchel et al. 2007). The increasing understanding of key psychological factors in the perpetuation of pain and pain-related disability has resulted in multiple treatment modalities for musculoskeletal disorders, and the effectiveness of psychological approaches in the management of these disorders has been evaluated in numerous meta-analyses (see e.g., Ehde et al. 2014; Guerrero Silva et al. 2018; Markozannes et al. 2017; Williams et al. 2012). Nonetheless, the effectiveness of psychological interventions is still inconsistent according to outcome research on return to work (RTW). The field of research on SA, i.e., insurance medicine, is fairly new and complex due to that the SA and RTW processes are influenced by a broad variety of incentives and risk factors (Alexanderson and Norlund 2004). To meet these needs, specific RTW interventions have been developed with the aim to specifically target workplace processes. Several meta-analyses have investigated the effects of these interventions.

With regard to musculoskeletal disorders, Meijer et al. (2005) found inconsistent results of interventions focusing on RTW for individuals on SA, but concluded that psychological treatment appeared to be an essential treatment component in interventions. On the other hand, Pike et al. (2016) found no advantage of psychological interventions over comparisons for chronic pain patients on work absence. In another systematic review multidisciplinary rehabilitation was found to be more effective for work outcomes compared with physical treatment, but not more effective than usual care (Kamper et al. 2015). However, a recent review of early multicomponent interventions for chronic pain suggested only limited effectiveness in reducing SA (Cochrane et al. 2017).

Turning to CMDs, Arends et al. (2012) found that problem-solving therapy (PST) for adults with adjustment disorder (i.e., stress-related disorders) enhanced partial RTW, but not full RTW, at 1-year follow-up. In the same systematic review, CBT did not reduce time to either partial or full RTW (Arends et al. 2012). For depressed workers, adding CBT to primary or occupational care reduced SA compared to usual care (Nieuwenhuijsen et al. 2014). Doki et al. (2014) divided studies into two groups. The first group consisted of studies with participants on SA at the time of randomization and the second group of studies included participants at risk for SA. There was no effect on RTW or SA duration for any of the groups compared to controls. However, when combining the two groups, there was a significant effect on SA duration (Doki et al. 2014). The lack of effect for the two groups separately might indicate a power problem. In another meta-analysis of interventions for targeting enhanced RTW for individuals with a CMD, the authors reported a modest effect on the reduced number of SA days but concluded that the available interventions did not lead to improved RTW rates compared to the control group (Nigatu et al. 2016).

In sum, so far there is no evidence of effectiveness of psychological treatment for either musculoskeletal disorders or CMDs on RTW, but there are some indications that psychological treatment may reduce SA days. Heterogeneity in pivotal factors such as metrics used, sample characteristics, and health and social insurance systems, is often discussed as a factor that complicates summary of the results as well as a general lack of eligible studies. Prior reviews have aimed at investigating subgroups to further understand the influence of these and other important factors. However, since too few trials have reported relevant data these analyses are lacking (Arends et al. 2012; Cochrane et al. 2017; Kamper et al. 2015), or studies may have been under-powered, and therefore, yielded non-significant effects (Nieuwenhuijsen et al. 2014).

So far, the content of RTW interventions differs greatly between trials. Evaluating SA presents considerable challenges at it is multifactorial and with complex roots. Which key mechanisms to target in RTW-focused interventions are still to a large extent unclear (Meijer et al. 2005). With regard to musculoskeletal disorders, the most common intervention seems to be based on team efforts, combining the expertise of different professions such as physician, occupational therapist, physical therapist and psychologist or social worker. For interventions targeting CMDs with a focus on RTW, the course of development in terms of treatment seems to be different. Treatments for CMDs are more often unimodal with a single professional responsible for the treatment (Blonk et al. 2006; van der Klink et al. 2003). The extent to which there is a focus on RTW and the inclusion of workplace interventions varies extensively in treatment protocols for both musculoskeletal disorders and CMDs.

Musculoskeletal disorders and CMDs are the most common diagnoses for individuals on SA. The low power in prior reviews, due to a scarcity of randomized controlled trials investigating SA and RTW, and the possibility of similar mechanisms involved in RTW for musculoskeletal disorders and CMDs, justifies an overall systematic synthesis of existing studies. Further, few prior meta-analyses on RTW interventions have investigated potential moderators of outcome. It is also important to be able to match effective treatment programs for different client populations to advance the development of the field in terms of more specific treatment guidelines.

The objectives of this systematic review and meta-analysis are to:

- Examine randomized controlled trials for the effectiveness of psychological interventions in reducing SA in patients on SA due to CMDs or musculoskeletal disorders compared to a waitlist control group, usual care or another clinical intervention.
- Evaluate possible differences in effectiveness of these interventions for patients with CMDs and musculoskeletal disorders.
- Investigate moderating factors such as background variables and treatment-specific variables on RTW.

## Method

### Eligibility criteria (PICOS)

#### Population

All studies of working age adults (18–65 years) on SA due to CMDs (i.e., mild to moderate symptoms of depression and anxiety disorders or symptoms related to conditions related to stress such as adjustment disorder or burnout) or musculoskeletal disorders were included in the review. Employment was not a requirement; unemployed on sickness benefits, and self-employed were also included. Exclusion criteria included studies focusing on participants with severe mental disorders such as psychosis, bipolar disorder, and substance abuse. Studies including participants with secondary pain due to malign illnesses or pain related to a prior accident were also excluded.

#### Interventions

All types of psychological interventions or psychotherapy were included. Psychological interventions were defined as being based on a psychological model or theory where qualified clinicians or treatment personnel deliver the treatment. Examples of therapies included are problem-solving therapy (PST), cognitive behaviour therapy (CBT), psychodynamic therapy (PDT), Multimodal Cognitive Behavioural Therapy (MMCBT), and Motivational Interviewing (MI). All types of psychological interventions were included if they were based on psychological theory and the purpose was to influence psychological processes with the aim to increase function or decrease symptoms. Interventions that did not have a coherent theoretical base, e.g., coaching, were excluded.

#### Controls

All control conditions were accepted, including psychological or non-psychological treatments, treatment as usual, pharmacological treatment, and waitlist. When there was more than one psychological treatment and a non-psychological treatment, all psychological treatments were compared with the non-psychological treatment as control condition. If a psychological treatment was compared to another psychological treatment within the same study, the experimental treatment and control group as chosen by the authors of that study were considered active treatment and control group, respectively.

#### Outcome measures

The primary outcome was time on SA, RTW, or increased working hours. There are many definitions of absence from work due to sickness. The present meta-analysis defines outcomes as fitting at least one of the following categories: time until first RTW, time until full RTW, cumulative duration of SA, i.e., total days of SA during the follow-up period (can be due to one or more SA spells), recurrence of SA (time in number of days until a recurrence or number of recurrences during follow-up), increased working hours, and time on disability pension. Data could either be presented as means and standard deviations (continuous) or as event data (categorical). Secondary measures of symptoms of depression, anxiety and stress were also included.

#### Study design

All randomized controlled trials (RCTs) including psychological interventions where an outcome of RTW or SA is included.

### Literature search

An extensive search was conducted in the following databases: Medline (Ovid), Web of Science Core Collection, Scopus, PsycInfo (Ovid), and PubMed until 2017-03-06. The initial search was conducted 2014-12-18 and the final search strategy was updated at two time points (2016-10-21, and 2017-03-06). Search strategies for the different databases are presented in Online Appendix 1.

#### Other resources

We also searched reference lists of other reviews and eligible studies. In some cases where data were missing in otherwise eligible studies, the authors were contacted to determine if complete data were available.

### Study selection

**Fig. 1:**
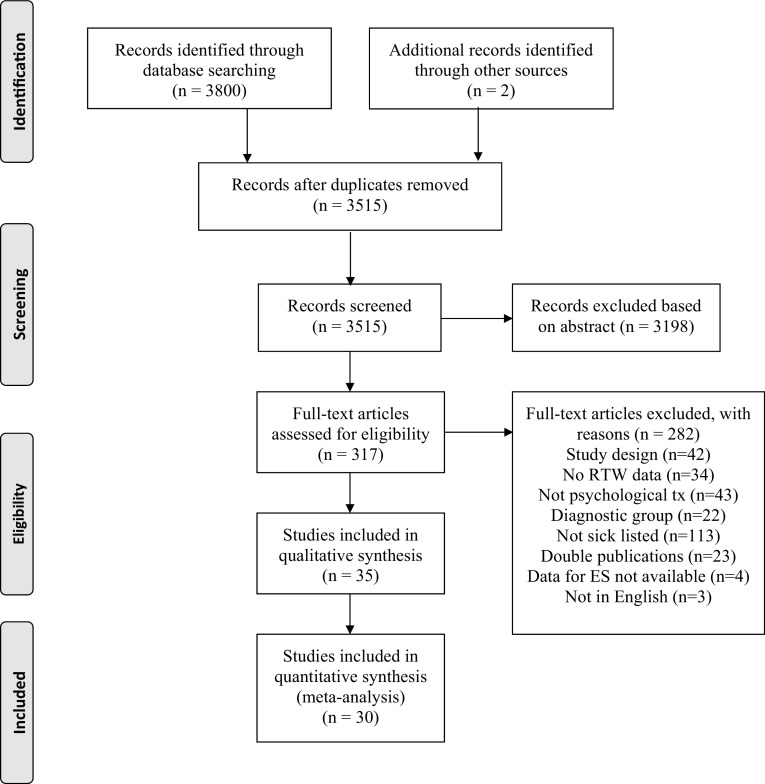
Flowchart of the inclusion of studies

Titles and abstracts of studies identified were stored in a database. Duplicates were removed and a bibliography including title and abstract was created. The study selection was completed in two steps. First, two authors independently screened titles and abstracts of all references to determine if each study met the inclusion criteria (AF reviewed all studies and the other co-authors reviewed a subdivision of studies each). A standardized digital form with inclusion criteria was used for this purpose and the inclusion criteria were: participants with CMDs or musculoskeletal disorders on SA and in working age, psychological intervention, and RCT. All the studies identified as possibly eligible in the first step were then fully reviewed a second time in full text format by two review authors (AF and PE), and subsequently assessed for inclusion and methodological quality. Exclusion criteria (population, intervention, outcome and design) were documented for each excluded study throughout the entire inclusion process. Figure 1 shows a flowchart of the inclusion of studies in the present meta-analysis, conducted according to the PRISMA criteria (Liberati et al. 2009).

### Data extraction

The first author extracted the data into an extraction form including essential study information, interventions, results on outcome measures, and data on moderator variables. These data were then double-checked by the second review author (PE). When there were disagreements about the data extraction, consensus was achieved by discussion. Since there were extensive heterogeneity in how studies reported SA, many studies were discussed. When no solution on how to extract data was achieved, e.g., due to missing data for the calculation of effect sizes, the study was excluded (see flowchart, Fig. 1).

### Categorization of potential moderators

Two categories of moderators were investigated; categorical and continuous. Categorical moderators included factors related to the intervention and study context. Continuous moderators included patient demographics and methodological quality of the studies. Moderators are further described below.

#### Diagnostic group

Study populations were categorized as CMD (i.e., depression, anxiety or stress-related ill health), musculoskeletal disorders, or CMD and musculoskeletal disorders.

#### Diagnosis

Study populations were categorized as depression, adjustment disorders, musculoskeletal disorder, CMD or musculoskeletal disorder, and CMD when there was a mix of mental health disorders in the sample.

#### Sickness absence duration

The number of weeks of continuous SA before randomization was noted for each study.

#### Type of treatment

The various psychological interventions were categorized into five subcategories: CBT (various types of CBT not specifically targeting the work situation), W-CBT (the treatment manual specifically targets RTW or work processes), PST, SFT, and MMCBT including interventions by at least two different professional categories. Control conditions were categorized as psychological interventions (if not the experimental condition in the trial), non-psychological interventions, treatment as usual (TAU) or waitlist (WLC).

#### Therapist profession

The professions of the therapists were categorized as occupational physician (including labour expert), psychologist (including psychotherapist), multimodal team (consisting of at least two professional categories), or other (including other mental health workers, social workers, stress management consultants, postgraduates, physical therapists, behaviour therapists, and one study where therapist profession was not specified).

#### Setting

Treatment setting was categorized as occupational health service, primary care, rehabilitation centre, and university.

#### Attrition

Participants who participated in at least one session but dropped out before treatment completion were counted as dropouts. In studies where the number of participants starting treatment was not reported, dropouts were counted from the number of participants randomized to treatment.

#### Other treatment-specific moderators

Several clinically justified moderators concerning the nature of the treatment were specified and categorized for each study. Duration was counted as the number of weeks that the intervention lasted (if there was no pre-defined intervention time, the number of weeks was used). The number of sessions, total treatment time (hours), intensity (hours per week), and booster sessions (Yes/No) was specified. Further, it was noted whether the intervention included workplace interventions (Yes/No) and if there was a clear work-focus, i.e., the full extent of the treatment protocol was tailored to target work or RTW (Yes/No). Whether the study evaluated therapist adherence to treatment protocol (Yes/No) and therapist competence (Yes/No) was also noted. Statistical analysis was categorized as intention-to-treat (ITT) if all randomized participants were included in the analyses and as completer analysis if dropouts were excluded. Year of publication and country of origin was noted for each study.

#### Methodological quality

The psychotherapy outcome study methodology rating scale (Öst 2008) was created with the aim of allowing for a wider range of scores than what was offered by prior RCT methodology scales. The scale consists of 22 items which are displayed in Table 1.

**Table 1:** Items of the psychotherapy outcome study methodology rating scale

1.	Clarity of sample description
2.	Severity/chronicity of the disorder
3.	Representativeness of the sample
4.	Reliability of the diagnosis in question
5.	Specificity of outcome measures
6.	Reliability and validity of outcome measures
7.	Use of blind evaluators
8.	Assessor training
9.	Assignment to treatment
10.	Design
11.	Power analysis
12.	Assessment points
13.	Manualized, replicable, specific treatment programs
14.	Number of therapists
15.	Therapist training/experience
16.	Checks for treatment adherence
17.	Checks for therapist competence
18.	Control of concomitant treatments
19.	Handling of attrition
20.	Statistical analyses and presentation of results
21.	Clinical significance
22.	Equality of therapy hours (for non-WLC designs only)

Two items, *5. Specificity of outcome measures*, and *6. Reliability and validity of outcome measures* were adapted for evaluating measures on SA/RTW. For specificity, measures on incidence were regarded as poor, time to event as fair, and continuous measures such as mean SA days or number of working hours or recurrent SA days were regarded as good. This categorization was based on the notion that continuous data lose specificity when it is dichotomized, hence provides less information compared to continuous data. This may be important specifically for SA data where the sum of SA days can be regarded as a more specific measure rather than the incidence of SA at a certain follow-up point taking into consideration the possible variability of SA status during the follow-up period. For reliability and validity self-reported data was regarded as fair and registry data as good.

Each item is rated as 0 = poor, 1 = fair, 2 = good, allowing for a range of 0–44 points. The internal consistency of the scale was acceptable with a Cronbach’s *α* = 0.622. The inter-rater reliability for the scale (between the first and second author) based on a random selection (20%) of the studies was ICC(2, 1) = 0.87 for the total score indicating a good overall inter-rater reliability.

### Meta-analysis

In the present meta-analysis, data from the retrieved RCTs were used to calculate effect size (ES) and to perform a meta-analysis on the continuous outcomes (SA days, increased working hours, etc.) and proportions of participants that successfully had achieved either partial of full RTW. The data were pooled with the software Comprehensive Meta-Analysis (CMA), version 2.3 which was used for all analyses. Since it cannot be expected that all effect sizes from the included studies come from the same population of effect sizes (because of the heterogeneity in the type of work disability, duration of SA, and the variation in interventions among studies), we employed a random effect model to compute the effect sizes. The results of each RCT were plotted as point estimates with corresponding 95% confidence intervals (CIs). Most RTW results were reported as time-to-event data (SA days or time until partial or full RTW). Means and associated standard deviations (SDs) were extracted for the cumulative duration of SA and for secondary outcomes levels of depression, anxiety, and stress symptoms. The ES was calculated as (*M*_intervention_ − *M*_control_)/SD_pooled_ for post- and follow-up assessments. Since there was no pre-defined post-assessment in a large proportion of the included studies, the mean of all follow-up assessment points was used to calculate ESs. Additionally, in case of more than one effect measure, the mean of these was used for each study. Each study contributed with an average of 2.6 ESs for continuous measures and 2.5 ESs for categorical measures (all measurement points combined). Before pooling the ESs the dataset was screened for statistical outliers. Instead of deleting outliers, they were replaced following the principles of Winsorizing (Lipsey and Wilson 2001) by reducing them to the exact value of *M* + 2SD. There were seven (6%) and four (8%) outliers replaced in the datasets with continuous variables and categorical variables, respectively.

Hedges’ *g* was computed to correct for small sample sizes. Values between 0.20 and 0.49 represent small ES, values between 0.50 and 0.79 are considered moderate ES, and values of 0.80 or higher represent large ES (Cohen 1988). For data on the number of events, odds ratios (OR) were computed. Values from 1.5 were interpreted as a small effect, 2.5 as a moderate effect and 4 as a large effect (Rosenthal 1996). The heterogeneity of the ES’s was calculated based on the *Q*-statistic (heterogeneity in ESs beyond random error) and the *I*-squared statistic (the percentage of the observed variance that shows actual differences in ESs between studies). Values above 75% indicate high heterogeneity, 50% medium heterogeneity, and 25% low heterogeneity (Higgins et al. 2003). Publication bias was assessed by examining the funnel plot on primary outcome measures, with the trim-and-fill method of Duval and Tweedie (2000) and Eggers’s regression intercept (Egger et al. 1997). Moderator analyses of continuous variables on which at least 75% of the studies provided information, were carried out with the meta-regression module in CMA (fixed effects model). For categorical variables, sub-group analysis using the mixed effects model was applied to assess moderation. If there were less than two studies in any condition being compared, the studies in that condition were excluded. Cochran’s *Q* (*Q*_between_) was computed to verify whether subgroups of treatments had identical effects. Statistical significance was defined as *p* < 0.05.

## Results

### Literature search and study selection

We retrieved 3513 peer-reviewed papers from 5 major databases. After screening the abstracts, 315 full-text articles were read and those that did not meet inclusion criteria (see flowchart Fig. 1 for a description of inclusion of the studies) were removed leaving a total of 30 studies (26 RCTs and 4 cluster RCTs) for inclusion. Table 2 describes the overall characteristics of the 30 trials. The included studies were published between 1992 and 2017 and included a total of 4024 participants in the meta-analysis. The mean number of participants per study was 134 (median 125.5, range 20–469). When one outlier was deleted (Haldorsen et al. 1998), the mean number of participants was 123. The most common country of origin was the Netherlands (13), followed by Sweden (7) and Norway (3).

**Table 2:** Background data for the included studies

Study	Country	Disorder	Diagnosis	*N*	Percent females	Mean age	SA duration mean (weeks)	SA inclusion criterion (weeks)^a^
Arends et al. (2014)	Netherlands	CMD	Common mental disorders	158	59	42	16.5	2–52
Beck et al. (2015)	Denmark	CMD	Stress	20	80	45	15.0	< 36
Bee et al. (2010)	USA	CMD	Mild/moderate mental health difficulties	53	49	45		1–13
Blonk et al. (2006)	Netherlands	CMD	Adjustment disorder	122	19	42	3.0	
Brouwers et al. (2006)	Netherlands	CMD	Minor mental disorders	194	59	40		< 12
de Vente et al. (2008)	Netherlands	CMD	Stress	82	39	41	9.0	2–26
de Weerd et al. (2016)	Netherlands	CMD	Common mental disorders	60	47	40	5.9	
Folke et al. (2012)	Sweden	CMD	Depression and unemployment	34	88	43	50.1	
Heiden et al. (2007)	Sweden	CMD	Stress	75	80	44	34.7	4–104
Kröger et al. (2015)	Netherlands	CMD	Depression	26	31	42		
Netterstrom et al. (2013)	Denmark	CMD	Stress	198	82	44	10.0	1–64
Noordik et al. (2013)	Netherlands	CMD	Common mental disorders	160	70	45	5.0	2–8
Stenlund et al. (2009)	Sweden	CMD	Burnout	136	71	42	47.9	12–104
van der Klink et al. (2003)	Netherlands	CMD	Adjustment disorder	192	37	40	2.0	> 2
Vlasveld et al. (2013)	Netherlands	CMD	Depression	126	54	43		4–12
Volker et al. (2015)	Netherlands	CMD	Common mental disorders	220	59	44	10.3	4–26
Altmaier et al. (1992)	USA	MD	Low back pain	45	27	40		12–120
Busch et al. (2011)	Sweden	MD	Nonspecific spinal pain	214	55	43		4–24
Haldorsen et al. (1998)	Norway	MD	Muscle pain	469	64	43	8.0	8–8
Heinrich et al. (2009)	Netherlands	MD	Musculoskeletal disorders	151	8	45	10.0	5–14
Leon et al. (2009)	Spain	MD	Musculoskeletal disorders	181	77	45	4.0	4–8
Lindell et al. (2008)	Sweden	MD	Back and neck pain	125	54	43		6–104
Marhold et al. (2001)	Sweden	MD	Musculoskeletal disorders	72	100	46	104.0	> 52
Meijer et al. (2006)	Netherlands	MD	Musculoskeletal disorders	38	68	38		4–20
Reme et al. (2016)	Norway	MD	Low back pain	203	55	45		8–40
Schiltenwolf et al. (2006)	Germany	MD	Low back pain	64	44	36	8.0	3–12
van den Hout (2003)	Netherlands	MD	Low back pain	84	24	41	8.6	< 20
Lytsy et al. (2017)	Sweden	CMD/MD	Mental health- or musculoskeletal disorder	206	100	49	388.0	
Nystuen and Hagen (2003)	Norway	CMD/MD	Mental health- or musculoskeletal disorder	213	59	40		> 7
Nystuen and Hagen (2006)	Norway	CMD/MD	Mental health- or musculoskeletal disorder	103	76	38		> 7

*CMD* common mental disorders, *MD* musculoskeletal disorders

^a^SA inclusion criterion describes the time on SA that was permitted for inclusion for the studies where this information was provided

### Participants: disorders and duration of sickness absence

Sixteen of the studies included participants with CMDs (depression = 3, stress disorders = 7, mixed mental disorders = 6), and 11 studies included participants with long-lasting musculoskeletal disorders. Three studies included participants with mental and/or musculoskeletal disorders. The mean proportion of females was 57.8% (range 8–100%). The mean age in the studies was 42.2 years (range 35.8–48.5). When two outliers were deleted (Schiltenwolf et al. 2006; Lytsy et al. 2017), the mean age was 42.4. Only 19 of the 30 studies had information on duration of SA at pre-treatment and the mean was 39 weeks (median 10, range 2–388). With one outlier deleted (Lytsy et al. 2017), the mean was 20 weeks. The mean attrition rate was 12.6% (median 10, range 0–38.5). When one outlier was deleted (Vlasveld et al. 2013), the mean was 11.6%.

### Treatment data

Table 3 describes the treatment data for the included studies. The methods of psychological treatments in this body of studies were diverse but most were based on CBT. The format of treatment was individual therapy (*n* = 18), group therapy (*n* = 8), group therapy combined with individual therapy (*n* = 3), and in one study there was a choice between group and individual therapy. The studies included CBT-based treatments (*n* = 22), including work-focused CBT (W-CBT = 3), and multimodal CBT (MMCBT = 3), cognitive therapy (CT = 3), acceptance and commitment therapy (ACT = 2), stress management therapy (SMT = 1), web-based CBT (ICBT = 1), exposure therapy (1), and mindfulness-based stress reduction therapy (MBSR = 1). Problem-solving therapy (PST) was applied in four studies and two studies used solution-focused therapy (SFT). One single study evaluated an intervention consisting of guided imagery and music (GIM). The profession of the therapists was reported in all studies but one, and the most common profession was psychologist (*n* = 8), followed by occupational physician (*n* = 5), multimodal team (*n* = 5), physical therapist (*n* = 2), psychotherapist (*n* = 1), and social worker (*n* = 1). Some studies used a mixture of professions such as health-care personnel (*n* = 1), mental health workers (*n* = 1), and either psychologist or occupational physician (*n* = 1). Other professions were stress management consultant (*n* = 1), labour expert (*n* = 1), and behaviour therapist (*n* = 1). Two studies used students as therapists (master students = 1, postgraduate students = 1). The duration of therapy was reported in 83% of the studies and the mean was 12 weeks (median 10, range 3–52). When two outliers were deleted (Lindell et al. 2008; Stenlund et al. 2009), the mean was 9.0 weeks.

**Table 3:** Treatment data for the included studies

Study	Method	Method category^a^	Format	Tx weeks	Tx sessions	Tx time (h)	Intensity (h/week)	Attri–ion %	Booster	FU (months)	WPI	Work-focus	Adherence	Comparison
Arends et al. (2014)	PST	PST	I	12	5	2.5	0.2	11.3	No	9	Yes	Yes	No	TAU
Beck et al. (2015)	GIM	GIM	I	9	6			8.3	No	6	No	No	No	TAU, WLC
Bee et al. (2010)	CBT	CBT	I	12	4.5	2.1	0.2	17.4	No	0	No	No	No	TAU
Blonk et al. (2006)	W-CBT	W-CBT	I	3	6	6	2	10.0	No	9	Yes	Yes	No	CBT^c^, TAU
Brouwers et al. (2006)	PST	PST	I	10	5	4.2	0.4	6.3	No	15	No	Yes	Yes	TAU
de Vente et al. (2008)	SMT	CBT	I	16	12	12	0.8	3.6	No	6	No	No	Yes	SMT group^c^, TAU
de Weerd et al. (2016)	W-CBT	W-CBT	I	4	7			9.7	No	0	Yes	Yes	No	W-CBT + CD^c^
Folke et al. (2012)	ACT	CBT	G	6	6	16	2.7	12.5	No	18	No	No	No	TAU
Heiden et al. (2007)	CT	CBT	G	10	20	60	6	28.6	Yes	12	No	No	No	PT^b^, TAU
Kröger et al. (2015)	W-CBT	W-CBT	I	24	22			0.0	Yes	12	No	Yes	No	CBT^c^
Netterstrom et al. (2013)	MBSR	CBT	G + I	12	16	24	2	4.8	No	0	Yes	Yes	No	TAU, WLC
Noordik et al. (2013)	Exposure	CBT	I		3.9				No	9	Yes	Yes	No	TAU
Stenlund et al. (2009)	CBR	CBT	G	52	80	140	2.7	6.5	Yes	36	No	No	No	Qigong + work rehab^b^
van der Klink et al. (2003)	CBT	CBT	I	6	5	1.5	0.3	22.9	No	10.5	Yes	No	No	TAU
Vlasveld et al. (2013)	PST	PST	I	12				38.5	No	9	Yes	No	No	TAU
Volker et al. (2015)	ICBT	CBT	I					10.0	No	9	No	Yes	No	TAU
Altmaier et al. (1992)	MMCBT	MMCBT	G + I	3					No	6	No	No	Yes	TAU
Busch et al. (2011)	MMCBT	MMCBT	G	4	20	34	8.5	22.2	Yes	120	Yes	No	Yes	CBT^c^, PT^b^, TAU
Haldorsen et al. (1998)	MMCBT	MMCBT	G + I	4	20	120	30	0.0	Yes	11	No	No	No	TAU
Heinrich et al. (2009)	CT	CBT	I	12	36	54	4.5	15.3	No	9	Yes	No	No	TAU
Leon et al. (2009)	CBT	CBT	I						Yes	6	No	No	No	TAU
Lindell et al. (2008)	CBT	CBT	I	42	37			1.6	Yes	0	Yes	No	No	TAU
Marhold et al. (2001)	CBT	CBT	G	12	12	30	2.5	5.6	Yes	6	No	Yes	No	TAU
Meijer et al. (2006)	CT	CBT	G	8	62	83	10.4	4.5	Yes	10	Yes	Yes	Yes	TAU
Reme et al. (2016)	CBT	CBT	I	10	9			23.3	Yes	9	No	No	Yes	Brief intervention^c^
Schiltenwolf et al. (2006)	CBT	CBT	I	3	15	90	30	3.0	No	23	No	No	No	TAU
van den Hout (2003)	PST	PST	G	8	28	33	4.1	22.4	Yes	12	No	No	No	Group education^c^
Lytsy et al. (2017)	ACT	CBT	I		10	10		15.7	No	0	No	No	No	TAU
Nystuen and Hagen (2003)	SFT	SFT	I/G		8	32		23.5	No		No	No	No	TAU
Nystuen and Hagen (2006)	SFT	SFT	G	8	8	32	4		No	10	No	No	No	TAU

*ACT* acceptance and commitment therapy, *ICBT* internet cognitive behaviour therapy, *CBT* cognitive behaviour therapy, *CD* convergence dialogue, *CT* cognitive therapy, *FU* follow-up, *G* group, *GIM* guided imagery and music, *I* individual, *MBSR* mindfulness-based stress reduction, *MMCBT* multimodal cognitive behaviour therapy, *PT* physical therapy, *PST* problem-solving therapy, *SFT* solution-focused therapy, *SMT* stress management training, *TAU* treatment as usual, *Tx* treatment, *W-CBT* work cognitive behaviour therapy, *WLC* waitlist control

^a^Method refers to how the intervention was described in each respective study and method category describes how the interventions were categorized in the active psychological treatment condition for this meta-analysis

^b^Categorized as non-psychological comparison group

^c^Categorized as psychological comparison group

The number of sessions was reported in 87% of the studies and the mean was 18 sessions (median 11, range 4–80). When two outliers were deleted (e.g., Stenlund et al. 2009; Meijer et al. 2006), the mean was 13 sessions. Treatment time in minutes was reported only in 67% of the studies and the mean total treatment time was 39 h (median 31, range 1.5–140). After deleting two outliers (Stenlund et al. 2009; Haldorsen et al. 1998), the mean total treatment time was 34 h (median 30). The mean follow-up time was 13.2 months (median 9, range 1–120). After deleting one outlier (Busch et al. 2011) the mean follow-up time was 9.4 months. Regarding work-specific treatment components, ten of the studies (33.3%) included a workplace intervention in the active treatment arm and 9 of the active treatment arms (30%) were work-focused, i.e., the interventions targeted mostly work-related processes. The methodological quality was rated in all studies. The average score was 17.0 (SD 3.6) with a range from 11 to 23.

### Control conditions

The different types of control groups were categorized into subgroups. First, another psychological treatment consisted of different types of CBT including W-CBT and SMT in groups. Non-psychological treatment included physical training, different types of physical therapy, graded activity, and Qigong. *TAU* control groups were the most common control alternative consisting of a variety of interventions such as occupational physician care according to guidelines or routine general practitioner care, physical therapy or vocational rehabilitation. Psychological treatments could be included in TAU in five studies as described by the authors but in some cases, the content in TAU was not specified. *Waitlist* controls were used in two studies.

### Primary outcome: sickness absence/RTW

Studies were only included if they reported on SA. Of the 30 included studies, 12 reported both continuous outcome measures (days to partial RTW or days to full RTW, increased working hours etc.), and categorical outcome measures (proportion of participants with partial RTW or full RTW). Nine studies reported only on days to RTW and another nine only on the proportion of participants with RTW.

#### Continuous outcomes

Table 4 shows the results for all assessment points based on the various types of comparisons for all studies and for studies on CMDs and musculoskeletal disorders respectively. For all studies, the overall ES was small (*g* = 0.16) but significantly different from zero. Heterogeneity was significant. When comparing the psychological studies with each control condition we found a small but significant effect size for treatment as usual (*g* = 0.13) and small but non-significant effect sizes when compared to psychological treatment (*g* = 0.21) and non-psychological treatment (*g* = 0.37).

**Table 4:** Effect sizes (Hedges’ *g*) for continuous measures of RTW divided on comparison conditions for all assessment time points

Comparison	*k*	*g* value	95% CI	*z* value	*Q* value	*I* ^2^
All psychological Tx studies	23	0.16	0.04 to 0.27	2.71^b^	41.2^b^	47
Psychological Tx vs. another psychological Tx	5	0.21	− 0.13 to 0.56	1.22	9.43	58
Psychological Tx vs. non-psychological Tx	1	0.37	− 0.03 to 0.78	1.81	0	0
Psychological Tx vs. TAU	17	0.13	0.004 to 0.25	2.03^a^	28.9^a^	45
Studies on CMDs only						
All psychological Tx studies	12	0.15	− 0.04 to 0.33	1.55	25.9^b^	57
Psychological Tx vs. another psychological Tx	3	0.03	− 0.59 to 0.65	0.09	7.24^a^	72
Psychological Tx vs. TAU	9	0.17	− 0.03 to 0.36	1.69	18.45^a^	57
Studies on musculoskeletal disorders only						
All psychological Tx studies	9	0.23	0.10 to 0.37	3.43^b^	5.86	0
Psychological Tx vs. non-psychological Tx	2	0.36	0.07 to 0.66	2.40^a^	0.01	0
Psychological Tx vs. TAU	6	0.16	0.00 to 0.32	1.97^a^	3.06	0

*Tx* treatment, *TAU* treatment as usual, *k* number of comparisons, *CI* confidence interval, *CMD* common mental disorder

^a^*p* < 0.05, ^b^*p* < 0.01

The studies were divided into two groups based on disorder type. Studies on CMDs showed small ESs for all comparisons (see Table 4) but none of these was significantly different from zero. Studies on musculoskeletal disorders showed a small overall ES (*g* = 0.23), a small ES when compared to non-psychological treatments (*g* = 0.36), as well as when compared with TAU (*g* = 0.16). These ESs were significantly different from zero.

##### Publication bias

The possibility of publication bias was investigated using Duval and Tweedie’s trim-and-fill method and Egger’s regression intercept. There was no problem with publication bias for the continuous outcome studies. Egger’s regression intercept was not significant (*p* = 0.590).

##### Moderator analyses

The following continuous variables were analysed with the meta-regression module in the CMA program using fixed effect analysis: number of participants in the trial, mean age of participants, proportion of females, duration of SA prior to randomization, attrition rate in the psychological treatment condition, treatment duration, number of sessions, total treatment time, treatment intensity, number of follow-up months, publication year, and methodological quality of the study. Two of these yielded a significant slope (see Table 5). Studies with longer duration of treatment were associated with lower ES for days on SA (*z* = − 2.64, *p* = 0.008). However, this ES was not significant when one study with the longest treatment duration (de Vente et al. 2008) was excluded from the analysis which suggests that this result is not robust. Further, for methodological quality, there was a significant slope where higher methodological scores were associated with higher ES (*z* = 3.04, *p* = 0.008).

**Table 5:** Meta-regression analyses (fixed effects) of the overall effect size of psychological treatment randomized controlled trials on sickness absence and return to work

Variable	*k*	Point estimate	*z* value	*p* value
*Continuous*				
*N*	23	0.0003	0.37	0.714
Age	22	0.006	0.27	0.788
% females	23	− 0.002	− 1.36	0.172
SA duration pre	13	− 0.0007	− 0.22	0.828
% attrition	20	0.008	1.36	0.172
Duration	17	− 0.036	− 2.64	0.008
# of sessions	19	0.0008	0.21	0.837
Treatment time	17	− 0.002	− 0.84	0.403
Intensity	15	0.015	0.89	0.372
FU months	18	0.012	1.01	0.312
Publication year	23	− 0.014	− 1.76	0.078
Methodology score	23	0.034	2.67	0.008
*Proportions*				
*N*	22	− 0.001	− 0.52	0.602
Age	22	− 0.052	− 0.92	0.356
% females	23	0.005	0.61	0.543
SA duration pre	16	− 0.013	− 1.35	0.176
% attrition	19	− 0.023	− 2.10	0.035
Duration	19	0.055	2.29	0.022
# of sessions	18	− 0.013	− 1.04	0.299
Treatment time	13	0.002	0.28	0.782
Intensity	13	− 0.136	− 1.73	0.084
FU months	23	− 0.012	− 0.84	0.400
Publication year	23	0.021	1.49	0.137
Methodology score	23	0.011	0.36	0.720

*FU* follow-up, *k* number of comparisons

For categorical moderator variables, sub-group analyses were employed in the CMA program (see Table 6 for results). Two moderator variables yielded significant *Q*_between_ values. If the treatment included booster sessions, the ES was larger, i.e., there were fewer days on SA, compared to when booster sessions were not included. There was a marginally significant difference in the format of the treatment. The group format resulted in higher ES than the individual format. However, it should be noted that all group therapies were conducted with musculoskeletal disorder patients.

**Table 6:** Subgroup analyses (mixed effects) of the overall effect size of psychological treatment randomized controlled trials for continuous outcomes on sickness absence and return to work

Variable	*k*	*g*	95% CI	*Q*_b_ value	*p* value
Disorder type				4.132	0.127
CMDs	12	0.147	− 0.04 to 0.33		
Musculoskeletal disorder	9	0.233	0.10 to 0.37		
CMDs and musculoskeletal disorders	2	− 0.085	− 0.36 to 0.19		
Diagnosis				4.488	0.213
Musculoskeletal disorder	9	0.233	0.10 to 0.37		
Adjustment disorder	6	0.199	− 0.11 to 0.51		
CMDs	5	0.079	− 0.21 to 0.36		
CMDs and musculoskeletal disorders	2	− 0.085	− 0.36 to 0.19		
Type of treatment				3.838	0.279
CBT	12	0.116	− 0.04 to 0.27		
W-CBT	4	0.224	− 0.20 to 0.64		
MMCBT	3	0.393	0.16 to 0.63		
PST	3	0.167	− 0.03 to 0.36		
Therapist profession				6.514	0.089
Multimodal team	5	0.265	0.07 to 0.46		
Occupational physician	4	0.157	− 0.12 to 0.43		
Psychologist/psychotherapist	7	− 0.044	− 0.24 to 0.15		
Miscellaneous	7	0.270	0.06 to 0.48		
Format				3.838	0.050
Group	6	0.363	0.17 to 0.55		
Individual	16	0.131	− 0.001 to 0.26		
Setting				5.028	0.081
Occupational health service	11	0.151	− 0.06 to 0.36		
Rehab centre	8	0.226	0.09 to 0.36		
University clinic	3	− 0.073	− 0.30 to 0.15		
Booster				4.820	0.028
No	17	0.102	− 0.03 to 0.24		
Yes	6	0.363	0.17 to 0.55		
Work place intervention included				0.200	0.655
No	13	0.180	0.03 to 0.33		
Yes	10	0.13	− 0.06 to 0.31		
Work focus				0.016	0.898
No	14	0.163	0.02 to 0.31		
Yes	9	0.147	− 0.05 to 0.35		
Adherence				0.087	0.768
No	16	0.149	0.01 to 0.29		
Yes	7	0.186	− 0.02 to 0.39		
Analysis				0.049	0.825
Completer	8	0.126	− 0.14 to 0.40		
Intent to treat	15	0.160	0.04 to 0.28		
Randomization				0.000	0.997
Cluster	3	0.154	− 0.22 to 0.53		
Patient	20	0.153	0.03 to 0.27		
Country				0.278	0.598
Netherlands	14	0.138	− 0.01 to 0.29		
Sweden	6	0.198	0.03 to 0.36		

*CBT* cognitive behaviour therapy, *CI* confidence interval, *CMD* common mental disorder, *k* number of comparisons, *g* Hedges g, *MMCBT* multi modal cognitive behaviour therapy, *PST* problem-solving therapy, *Q*_*b*_*Q* between subgroups, *W-CBT* work cognitive behaviour therapy

#### Categorical outcomes: proportions of participants with partial or full RTW

Table 7 displays the results on RTW for all studies and all measurement points and various types of comparisons. For the categorical outcomes, there was a small overall ES (OR 1.43) for psychological treatments, which was significantly different from zero. Heterogeneity was significant. The effect size for psychological treatment compared with TAU (OR 1.47) was also significantly different from zero, and heterogeneity was significant. Compared with other psychological control conditions (OR 1.12), non-psychological treatments (OR 0.89), and waitlist controls (OR 4.43), the ES’s were not significantly different from zero and there was no significant heterogeneity.

**Table 7:** Effect sizes (odds ratio) for proportions of RTW divided on comparison conditions for all assessment time points

Comparison	*k*	OR	95% CI	*z* value	*Q* value	*I* ^2^
All psychological Tx studies	23	1.43	1.06–1.92	2.36^a^	47.71^b^	54
Psychological Tx vs. another psychological Tx	5	1.12	0.52–2.42	0.30	8.62	54
Psychological Tx vs. non-psychological Tx	2	0.89	0.43–1.84	− 0.32	0.06	0
Psychological Tx vs. TAU	14	1.47	1.06–2.05	2.28^a^	25.78^a^	50
Psychological Tx vs. WL	2	4.43	0.89–22.08	1.82	1.70	41
*Studies on CMDs only*						
All psychological Tx studies	15	1.67	1.15–2.41	2.72^b^	23.64	41
Psychological Tx vs. another psychological Tx	2	2.46	0.99–6.10	1.94	0.82	0
Psychological Tx vs. non-psychological Tx	2	0.89	0.43–1.84	− 0.32	0.06	0
Psychological Tx vs. TAU	9	1.54	1.02–2.31	2.06^a^	11.97	33
Studies on musculoskeletal disorders only						
All psychological Tx studies	7	1.03	0.64–1.65	0.13	14.67^a^	59
Psychological Tx vs. another psychological Tx	2	0.59	0.35–0.99	− 2.00^a^	0.14	0
Psychological Tx vs. TAU	4	1.34	0.69–2.58	0.86	10.32^a^	71

*CI* confidence interval, *CMD* Common mental disorder, *k* number of comparisons, *TAU* treatment as usual, *Tx* treatment, *WL* waitlist

^a^*p* < 0.05, ^b^*p* < 0.01

For CMDs only, there was an overall small ES (OR 1.67) that was significantly different from zero. When compared with TAU there was again a small ES (OR 1.54) which was significantly different from zero. For studies on musculoskeletal disorders there was a significant ES when compared with other psychological treatments (OR 0.59), but not when compared with TAU.

##### Publication bias

There was some indication of a risk of publication bias for the categorical outcomes of RTW. Regarding the overall ES, the trim-and-fill method suggested that 8 studies should be trimmed. Egger’s regression intercept also yielded a significant *t* value (*t* = 2.290; *p* = 0.032).

##### Moderator analyses

The same continuous moderator variables as for the continuous outcome measures were analysed for the categorical outcomes (see Table 5). Studies with a higher proportion of attrition were associated with lower ES, that is, less RTW. For the duration of treatment, longer treatments were associated with higher ES. However, when one study with the longest treatment duration (Kröger et al. 2015) was excluded from the analysis, this ES was no longer significant suggesting that this result is not robust. For categorical outcomes, there was no moderation of methodological quality.

Five of the categorical moderator variables yielded significant *Q*_between_ values (see Table 8). There was a significant difference between different types of professionals giving the treatment. Treatment delivered by psychologists/psychotherapists and occupational physicians was associated with larger ES. Studies conducted within university departments also yielded higher ES compared to occupational health services and rehab centres. Including booster sessions was associated with lower ES compared to not including booster sessions. Having a work-focus in the treatment manual yielded larger ES. Finally, there was a significant difference between different countries; studies from Denmark yielded higher ES than studies from the Netherlands, Norway and Sweden.

**Table 8:** Subgroup analyses (mixed effects) of the overall effect size of psychological treatment RCTs for proportions of RTW

Variable	*k*	OR	95% CI	*Q*_b_ value	*p* value
Disorder type				2.489	0.115
CMDs	15	1.667	1.15–2.41		
Musculoskeletal disorder	7	1.031	0.64–1.65		
Diagnosis				2.734	0.434
Adjustment disorder	9	1.815	0.94–3.52		
Depression	3	1.594	0.84–3.04		
CMDs	3	1.436	0.93–2.21		
Musculoskeletal disorders	7	1.031	0.64–1.65		
Type of treatment				4.808	0.186
CBT	13	1.586	0.95–2.66		
W-CBT	2	1.969	0.71–5.45		
MMCBT	2	0.902	0.62–1.31		
PST	4	1.403	0.92–2.15		
Therapist profession				14.458	0.002
Multimodal team	3	0.895	0.64–1.24		
Occupational physician	6	2.471	1.36–4.51		
Psychologist/psychotherapist	6	2.014	1.14–3.58		
Miscellaneous	8	0.860	0.60–1.24		
Format				1.338	0.512
Group	6	1.078	0.67–1.74		
Individual	13	1.410	0.99-2.00		
Group + individual	4	2.090	0.60–7.25		
Setting				14.663	0.001
Occupational health service	8	1.697	1.19–2.42		
Rehab centre	11	1.007	0.71–1.44		
University department	3	5.210	2.35–11.55		
Booster				11.677	0.001
No	14	2.024	1.35–3.03		
Yes	9	0.894	0.70–1.14		
Work place intervention included				3.648	0.056
No	17	1.099	0.83–1.45		
Yes	6	2.548	1.35–4.83		
Work focus				5.596	0.018
No	17	1.099	0.83–1.45		
Yes	6	2.548	1.35–4.83		
Adherence				3.296	0.069
No	18	1.620	1.15–2.29		
Yes	5	0.920	0.56–1.52		
Analysis				2.643	0.104
Completer	11	1.863	1.02–3.41		
Intent to treat	12	1.088	0.86–1.38		
Randomization				0.446	0.504
Cluster	3	1.711	1.04–2.82		
Patient	20	1.394	1.00–1.95		
Country				20.674	0.000
Denmark	3	5.684	2.67–12.10		
Netherlands	9	1.561	1.13–2.15		
Norway	3	0.962	0.56–1.66		
Sweden	5	0.823	0.52–1.30		

*CBT* cognitive behaviour therapy, *CI* confidence interval, *CMD* common mental disorder, *k* number of comparisons, *MMCBT* multi modal cognitive behaviour therapy, *OR* odds ratio, *PST* problem-solving therapy, *Q*_*b*_*Q* between subgroups, *W-CBT* work cognitive behaviour therapy

### Secondary outcomes: symptoms

Only 13 studies included data on symptoms of mental problems. The overall ES was 0.11 (*k* = 16, 95% CI − 0.008 to 0.22) for all assessment points, which was not significant from zero (*z* = 1.82, *p* = 0.068). Heterogeneity was not significant (*Q* = 12.72, *p* = 0.624). At post-assessment, the ES was 0.11 (*k* = 15, 95% CI − 0.04 to 0.27), also not significant (*z* = 1.40, *p* = 0.162), and heterogeneity was not significant (*Q* = 21.45, *p* = 0.091). For depression, the overall ES was 0.09 (*k* = 13, 95% CI − 0.05 to 0.22) for all assessment points and not significant (*z* = 1.30, *p* = 0.195). Likewise, for anxiety, there was no significant difference from zero for the overall ES 0.06 (*k* = 10, 95% CI − 0.09 to 0.12; *z* = 0.74, *p* = 0.459).

## Discussion

### Summary of evidence

This systematic review and meta-analysis identified 30 RCTs published from 1998 to 2017. With few exceptions, psychological treatments were based on cognitive-behavioural approaches whereas the content and forms of implementation varied extensively. The results showed a small but significant difference for primary continuous outcome measures in favour of the psychological treatments. There was also a small but significant difference compared with TAU but no significant differences when compared to other psychological or non-psychological (e.g., physical therapy, Qigong) control conditions. When psychological interventions are compared with the condition “another psychological treatment”, sometimes CBT is compared to CBT, and the difference between these intervention formats might be too small to generate a meaningful difference in effect. Noteworthy, there are fewer group comparisons for other clinical interventions, psychological or non-psychological, compared to comparisons including TAU. It cannot be excluded that the lack of significance may be due to lack of power in these cases. In evaluating proportions of partial or full RTW, there was also a small significant overall effect in favour of psychological treatments. Psychological treatment was significantly better than TAU (small ES), but not compared to the other active control conditions. Thus, overall, psychological treatments seem to have a small positive effect on RTW for patients on SA due to CMDs and/or musculoskeletal disorders. Previous meta-analyses have not been unanimous but rather have pointed in different directions. Altogether, the evidence so far suggests that even though there is a detectable advantage of psychological treatments on RTW, the effect is small and probably not of clinical significance. However, the results are inconclusive as to what the most effective form of psychological treatment is and most of the included studies did not specifically address RTW which implies extensive room for improvement of interventions in this field.

The effectiveness of psychological treatments was also examined for CMDs and musculoskeletal disorders separately. For both CMDs and musculoskeletal disorders, CBT was the most common intervention and TAU was the most frequent comparison group. For CMDs, there were no significant differences in ES for continuous measures, but there was a small significant ES overall and when compared to TAU for the categorical outcomes, i.e., proportions of partial or full RTW. For studies on musculoskeletal disorders there were significant ESs for the continuous outcomes, however, there was no true heterogeneity for these analyses, suggesting that these results are due to sampling error only. All in all, the results for studies on CMDs and musculoskeletal disorders separately shows no clear differences in primary outcomes as opposed to the main analysis where studies on mental disorders and musculoskeletal disorders were combined.

There was no significant overall effect on the secondary outcomes in terms of symptoms of depression and anxiety. Lack of differences between study groups on improved symptoms may indicate either that the experimental intervention and the control group were equally effective or that natural recovery has occurred in both groups, regardless of intervention. This raises the question whether it is enough if an intervention only yields a significant reduction of SA. Ideally, an effective psychological intervention for workers on SA due to CMDs should both reduce SA and improve symptoms. In some studies, RTW improved but not symptoms. In other studies symptoms improved but there was no evidence of improved RTW. Altogether, improved mental health may facilitate, but is not sufficient for successful RTW (Arends et al. 2014; Ejeby et al. 2014).

#### Moderators

##### Methodological quality

For continuous outcomes, a higher methodological score was associated with larger ESs. This finding is interesting and may be attributed to the high sensitivity of the scale used, Psychotherapy outcome study methodology rating scale (Öst 2008), with a theoretical range of 0–44 and an actual range of 11–23 in this study. This is in line with a previous meta-analysis of treatments of OCD in children where higher methodology quality also was associated with larger ES (Öst et al. 2016). However, the methodological quality score did not moderate the outcome for the categorical outcomes. This makes it difficult to draw firm conclusions about the significance of methodological quality for the outcome in this field in general. However, the methodological quality of a majority of included studies was low, which is not surprising given the risk of bias and methodological concerns discussed in previous meta-analyses in this field (Arends et al. 2012; Nieuwenhuijsen et al. 2014; Nigatu et al. 2016). Hence, we conclude that low methodological quality is a problem to address before further evaluation of evidence for psychological treatment for RTW is carried out.

##### Treatment-specific variables

For continuous measures, shorter treatment duration was associated with larger ES whereas, for the categorical RTW outcomes, longer treatments were associated with larger ES. However, this contradictive result may best be explained by the fact that two studies with the longest treatment periods had very different results and when excluded from the analysis (de Vente et al. 2008 for continuous outcomes and; Kröger et al. 2015 for dichotomous outcomes), the ESs were no longer significant. Shorter treatment duration has previously been associated with a higher effect size for psychological outcome variables examined as a moderator for stress management intervention programs (Richardson and Rothstein 2008), but this conclusion cannot be derived from the present meta-analysis.

The proportion of attrition also significantly moderated the effect sizes for the categorical outcomes: there was a negative slope suggesting that more attrition was associated with lower ES. This may be due to participants that RTW earlier, also tend to drop out to a larger extent. For three of the continuous moderator variables (SA duration pre, treatment time and treatment intensity), less than 75% of the studies provided information (see Tables 2, 3, 5). For this reason these analyses were disregarded since they cannot be considered as reliable due to missing data.

Sub-group analyses further revealed that including booster sessions generated higher ES compared to no booster session for continuous outcomes. Eleven trials included booster sessions of which eight trials were on musculoskeletal disorder patients. That is, 8 of 11 studies on musculoskeletal disorder patients included booster sessions. However, when analysed separately for CMDs and musculoskeletal disorders, there were no significant effects. Nonetheless, for categorical outcomes the moderating effect was in the other direction, the ES was larger when booster sessions were not included. These contradicting results may be due to differences in how booster sessions were implemented in different studies. Including booster sessions aims at sustaining treatment outcomes and preventing relapse and has been evaluated in a previous RCT on pain treatment where the beneficial effects failed to reach statistical significance (Mangels et al. 2009).

Further, four other categorical variables related to the design of the treatment protocol significantly moderated outcome for categorical outcomes on RTW, but not for continuous outcomes. First, the profession of the therapist moderated outcome. The highest ES was generated by occupational physicians (including labour experts) which may indicate that a deeper understanding of insurance medicine may beneficiate RTW. Additionally, psychologists/psychotherapists had a better outcome than other professions, indicating that a deeper understanding of psychological methods might improve outcome. This result is in line with previous meta-analyses (e.g., Öst and Ollendick 2017; Seekles et al. 2013). Since most musculoskeletal disorder studies involved various therapist professions in multimodal teams, this is applicable only to studies of CMD diagnoses.

Second, the setting in which the study was performed also moderated outcome for categorical outcomes. Studies carried out in university settings had higher ESs than those from occupational health services and rehab centres, which might be an indication of differences between efficacy and effectiveness.

Third, there was a significant effect of an inherent work-focus in the treatment protocol for categorical outcomes. This can only be said to be true for studies on CMDs since no musculoskeletal disorder trial included this in the study design. Additionally, for CMDs, there was a significant effect on including a workplace intervention. However, only one study on musculoskeletal disorders (Marhold et al. 2001) reported on proportions of RTW. Including a workplace intervention yielded a borderline significant moderating effect. In a previous meta-analysis, work-place interventions were favourable regarding RTW for disabled workers with musculoskeletal disorders, but not for disabled workers with CMDs (van Vilsteren et al. 2015). However, in this meta-analysis, the workplace interventions were merely one part of the interventions given and not a standalone intervention. In another meta-analysis, Nigatu et al. (2016) noted that most trials included did not specifically address RTW, but rather aimed at symptomatic improvement. While we included more trials than van Vilsteren et al. (2015) and Nigatu et al. (2016) in the present meta-analysis, we reached the same conclusion. Only 9 out of 30 trials comprised an experimental condition where RTW was essentially the target of intervention expressed in a treatment protocol specifically tailored for the aim of RTW. It was more common for interventions to primarily aim at reducing symptoms. The relation between symptomatic improvement and returning to work after SA has been raised earlier when symptom reduction was not accompanied by a reduction in SA (Ejeby et al. 2014). The current understanding of the RTW process highlights the need for involvement of work stakeholders and work-specific treatment components, also in line with various guidelines, e.g., NICE (2009).

Finally, there was a significant effect of the country where the trial was carried out for categorical outcomes. This variable is of special interest due to differences in the organization of the occupational health-care services in different countries. Studies from Denmark yielded the highest ESs. However, the Nordic countries are similar in SA policies and in this case, differences are probably due to study design. Three study groups originating from two Danish studies were included in this comparison and consisted of guided imagery and music therapy vs. waitlist control (Beck et al. 2015) and mindfulness-based stress reduction therapy vs. waitlist control and TAU (Netterström et al. 2013). Comparisons with waitlist control yielded the largest ESs and contributed to the larger overall ES for Danish studies. None of the Dutch, Norwegian or Swedish studies included a waitlist control. TAU and other active treatments were the most common comparison (see Table 3). Thus, this result is probably a result of weaker comparison groups in the Danish studies. Most studies originated from the Netherlands where sick-leave certification is entirely handled by occupational physicians, in contrast to the Nordic countries where non-specialized physicians also certify sick-leave. Insurance medicine is a field influenced by many factors ranging from the legislative and insurance system, workplace, healthcare to personal variables (Loisel and Anema 2013). The modest effects found from these treatments may be explained with a lack of work focus in the treatment protocols and the lack of involvement of more than one stakeholder.

### Publication bias

The analysis of publication bias suggests that for continuous outcomes this is not a problem for the current meta-analysis. For categorical outcomes on RTW; however, there is a problem regarding publication bias. These contradictory results indicate uncertainty regarding publication bias. Nonetheless, due to the loss of information when dichotomizing data, we give more weight to the continuous outcomes, and therefore, conclude that publication bias is probably not a problem for the current meta-analysis.

### Methodology

Some of the items in the methodology rating scale (Öst 2008) received consistently low ratings across the included trials. **‘**Reliability of the diagnosis in question**’**, is probably not as important in the SA field as in psychiatric disorders. An evaluation of the primary outcome **‘**sickness absence**’** is not always related to a specific diagnosis since sub-syndromal levels of symptoms also may be associated with SA. A weak point in the included trials is related to the assessment procedure. As noted by others previously (Alexanderson and Norlund 2004; Hensing 2004; van Poppel et al. 2002), the field struggles with inconsistencies in measurements and many different measures of SA and RTW were employed in the studies. Proportions of RTW, perhaps a less specific measure, could possibly overestimate the effect since it evaluates RTW status only at the assessment point. Days on SA might give an improved illustration of the amount of SA during the follow-up period. The reliability of how the measurements were implemented is reflected in the quality scale item **‘**Assessor training**’**, which was reported in only two trials. Further, only seven studies used blind evaluators. Another problem related to the assessment procedure in the included studies is the lack of measurement directly post-intervention, which is common practice in psychological intervention research to distinguish direct effects from follow-up effects.

Another problem area in these trials concerns the integrity of the treatments delivered. Only four trials reported that treatment adherence was assessed by checks of therapy tapes and no study reported on therapist competence. Although this is a labour-intensive and expensive part of the process, it is the only way to evaluate therapist fidelity to the treatment protocol. Therapist drift (gradually deviating from the treatment protocol) is a well-known phenomenon in psychotherapy research and needs to be addressed (Boswell et al. 2013) in clinical trials. Likewise, checks for therapist’s competence was completely lacking in this collection of trials. Further, checks for concomitant treatments were only made in five trials. Thus, the effects may at least partly, stem from other treatments that participants may have obtained during the intervention period.

Thus, the quality of study methodology needs to be improved. The methodological weaknesses contribute to an overall difficulty of drawing firm conclusions on the effectiveness of psychological treatments in this field. We cannot for instance properly evaluate whether the treatments have been delivered according to protocols and with sufficient therapeutic expertise to generate the expected outcome.

### Strengths and limitations

This meta-analysis includes only participants who were on SA at the time for inclusion in the study. Improving the situation for employees on SA may be difficult since previous long-term SA is a strong predictor of future SA (Hultin et al. 2012). Therefore, it is important to distinguish between patients already on SA from those at risk for SA. Ideally, patients on short SA should also be distinguished from patients on longer periods of SA. However, there are currently not enough trials to run these analyses with adequate power. There are some further limitations to this review. A meta-analysis is never better than the included studies and as noted in the methodological overview, there are some important methodological concerns in the included studies. The search was restricted to only peer-reviewed journals, excluding other sources which might include relevant studies. The tests that we used to investigate how much our results were potentially influenced by publication bias may not entirely capture this problem. Some studies may be undertaken as part of evaluating policy by policy-makers, which may be reported as part of governmental reports and never be considered for publication in peer-reviewed journals. However, it may be assumed that RCT’s generally are conducted within an academic research context where reports generally are published. Another previously mentioned limitation is the lack of consensus on measures in this field. The diverse set of measures presented in the trials makes it more difficult to draw strong conclusions on effect and compare outcomes from different studies. This constitutes a problem especially for continuous measures in this meta-analysis where different measures (e.g., days on SA, hours worked, increased work hours) are combined. Despite the limitations of this approach, we found that combining the measures was the most relevant way to analyse data to maximise power and not exclude studies due to a problem that is symptomatic for this research field. Another possible limitation is the inclusion of psychological interventions as a control condition. When this was the case, it was due to the fact that the trial fulfilled inclusion criteria and evaluated at least one psychological treatment, and the control condition consisted of another psychological treatment. To evaluate whether the effects differed depending on which control condition was included, we investigated different subgroups of control conditions, i.e., psychological, non-psychological, and TAU, separately. Finally, a potential threat to the quality of the evidence is the management of multiple trial arms and multiple outcome measures in this meta-analysis. We chose to include each trial arm in the comparison and outcome measures without taking into consideration the control group being used more than once for each study. This problem can best be handled using multilevel methods for meta-analysis, however, a larger number of studies is then necessary than what is included in this meta-analysis (Moeyaert et al. 2017). Since we could not find satisfactory methodological solutions for these problems, we recognize that the effect sizes should be interpreted cautiously.

## Conclusion

In conclusion, this review found some evidence supporting the effectiveness of psychological interventions for RTW for the most common diagnoses related to SA. However, the results also point to some variables to consider when designing future RTW treatment protocols, such as including workplace relevant components and work-focused interventions, as well as an overall high methodological quality. The results of this meta-analysis underline the assessment problem discussed in this field, and there is a need to agree on a valid method to assess RTW taking into consideration all essential aspects of the phenomenon and allowing for meaningful comparison between studies.

## Electronic supplementary material

Below is the link to the electronic supplementary material.

Supplementary material 1 (DOCX 20 KB)
