# Supplementary material for: Psychological treatments for return to work in individuals on sickness absence due to common mental disorders or musculoskeletal disorders: a systematic review and meta-analysis of randomized-controlled trials
Source: Int Arch Occup Environ Health. 2018 Nov 24;92(3):273–93. doi: 10.1007/s00420-018-1380-x (PMC6420449; doi:10.1007/s00420-018-1380-x)
Supplement: Supplementary file 1 — Supplementary material 1 (DOCX 20 KB) [file 420_2018_1380_MOESM1_ESM.docx]

Appendix 1: Documentation of search strategies

Databases:

1. Medline (Ovid)
2. Web of Science Core Collection
3. Psycinfo (Ovid)
4. Pubmed
5. Scopus

1. Medline

1. exp Anxiety Disorders/

2. Adjustment Disorders/

3. Anxiety/

4. Depression/

5. exp Mood disorders/

6. exp Stress, Psychological/

7. (anxiety* or affective* or bipolar* or mental problem* or mental disorder* or adjustment or depress* or stress* or burnout or panic* or phobic*).tw.

8. exp Pain/

9. Fibromyalgia/

10. Musculoskeletal Diseases/

11. (pain or fibromyalgia or neuralgia or myalgia or ache* or discomfort* or complaint* or musculo* or suffer* or nocicept*).tw.

12. 1 or 2 or 3 or 4 or 5 or 6 or 7 or 8 or 9 or 10 or 11

13. exp Psychotherapy/

14. Problem Solving/

15. exp Adaptation, Psychological/

16. exp Mind-Body Therapies/

17. Patient education as topic/

18. ((cognitive or behav* or (acceptance adj3 commitment) or psychodynamic or psychoanaly* or psychologic* or relax*) adj2 (therap* or treatment* or intervention*)).tw.

19. (psychotherap* or psychoeducat* or feedback* or (problem* adj2 solv*) or mindfulness).tw.

20. 13 or 14 or 15 or 16 or 17 or 18 or 19

21. Rehabilitation, Vocational/

22. Employment/

23. Return to Work/

24. Sick Leave/

25. Absenteeism/

26. ((sick* adj2 absen*) or work resumption or (work adj2 absen*) or medical leave or sick leave or absenteeism or (return* adj2 work) or ((vocation* or employment or work) adj2 rehabilit*)).tw.

27. 21 or 22 or 23 or 24 or 25 or 26

28. 12 and 20 and 27

29. remove duplicates from 28

30. limit 29 to yr="1980 -Current"

31. limit 30 to (danish or english or norwegian or swedish)

2. Web of Science Core Collection

TS=(anxiety* OR affective* OR bipolar* OR “mental problem*” OR “mental disorder*” OR adjustment OR depress* OR stress* OR burnout OR panic* OR phobic* OR pain OR fibromyalgia OR neuralgia OR myalgia OR ache* OR discomfort* OR complaint* OR musculo* OR suffer* OR nocicept*)

AND

TS=((cognitive OR behav* OR (acceptance NEAR/3 commitment) OR psychodynamic OR psychoanaly* OR psychologic* OR relax*) NEAR/2 (therap* OR treatment* OR intervention*) OR psychotherap* OR psychoeducat* OR feedback* OR mindfulness OR (problem* NEAR/2 solv*))

AND

TS=((sick* NEAR/2 absen*) OR “work resumption” OR (work NEAR/2 absen*) OR “medical leave” OR “sick leave” OR absenteeism OR (return* NEAR/2 work) OR ((vocation* OR employment OR work) NEAR/2 rehabilit*))

Refined by: LANGUAGES: ( ENGLISH ) AND PUBLICATION YEARS: ( 2016 OR 2006 OR 2001 OR 1996 OR 2015 OR 2017 OR 2004 OR 1994 OR 2014 OR 2008 OR 1997 OR 1992 OR 2013 OR 2005 OR 2002 OR 1995 OR 2011 OR 2010 OR 2000 OR 1993 OR 2009 OR 2003 OR 1998 OR 1991 OR 2012 OR 2007 OR 1999 )

Refined by: LANGUAGES: ( ENGLISH )

Indexes=SCI-EXPANDED, SSCI, A&HCI, CPCI-S, CPCI-SSH, ESCI Timespan=All years

3. Psycinfo

1. exp anxiety disorders/

2. anxiety/

3. exp adjustment disorders/

4. major depression/

5. exp Affective Disorders/

6. exp stress/

7. (anxiety* or affective* or bipolar* or mental problem* or mental disorder* or adjustment or depress* or stress* or burnout or panic* or phobic*).tw.

8. exp pain/

9. fibromyalgia/

10. exp musculoskeletal disorders/

11. (pain or fibromyalgia or neuralgia or myalgia or ache* or discomfort* or complaint* or musculo* or suffer* or nocicept*).tw.

12. 1 or 2 or 3 or 4 or 5 or 6 or 7 or 8 or 9 or 10 or 11

13. exp psychotherapy/

14. problem solving/

15. exp adjustment/

16. exp mind body therapy/

17. exp client education/

18. ((cognitive or behav* or (acceptance adj3 commitment) or psychodynamic or psychoanaly* or psychologic* or relax*) adj2 (therap* or treatment* or intervention*)).tw.

19. (psychotherap* or psychoeducat* or feedback* or (problem* adj2 solv*) or mindfulness).tw

20. 13 or 14 or 15 or 16 or 17 or 18 or 19

21. exp Vocational Rehabilitation/

22. employment status/

23. exp reemployment/

24. exp employee leave benefits/

25. exp employee absenteeism/

26. ((sick* adj2 absen*) or work resumption or (work adj2 absen*) or medical leave or sick leave or absenteeism or (return* adj2 work) or ((vocation* or employment or work) adj2 rehabilit*)).tw.

27. 21 or 22 or 23 or 24 or 25 or 26

28. 12 and 20 and 27

29. limit 28 to yr="1980 -Current"

30. limit 29 to (danish or english or norwegian or swedish)

4. Pubmed

((anxiety*[tiab] OR affective*[tiab] OR bipolar*[tiab] OR mental problem*[tiab] OR mental disorder*[tiab] OR adjustment[tiab] OR depress*[tiab] OR stress*[tiab] OR burnout[tiab] OR panic*[tiab] OR phobic*[tiab] OR pain[tiab] OR fibromyalgia[tiab] OR neuralgia[tiab] OR myalgia[tiab] OR ache*[tiab] OR discomfort*[tiab] OR complaint*[tiab] OR musculo*[tiab] OR suffer*[tiab] OR nocicept*[tiab])

AND

cognitive[tiab] OR behav*[tiab] OR (acceptance commitment[tiab]) OR psychodynamic[tiab] OR psychoanaly*[tiab] OR psychologic*[tiab] OR relax*[tiab] OR psychotherap*[tiab] OR psychoeducat*[tiab] OR feedback*[tiab] OR mindfulness[tiab])

AND

(“sickness absence”[tiab] OR “work resumption”[tiab] OR “medical leave”[tiab] OR “sick leave”[tiab] OR absenteeism[tiab] OR (vocation* rehabilit*[tiab]) OR (employment rehabilit*[tiab]) OR (work rehabilit*[tiab]) ))

NOT

medline[sb]

Filters: English; Norwegian; Danish; Swedish

5. Scopus

( TITLE-ABS-KEY ( **anxiety***  OR  **affective***  OR  **bipolar***  OR  **"mental problem*"**  OR  **"mental disorder*"**  OR  **adjustment**  OR  **depress***  OR  **stress***  OR  **burnout**  OR  **panic***  OR  **phobic***  OR  **pain**  OR  **fibromyalgia**  OR  **neuralgia**  OR  **myalgia**  OR  **ache***  OR  **discomfort***  OR  **complaint***  OR  **musculo***  OR  **suffer*** ) )

AND

( TITLE-ABS-KEY ( ( **cognitive**  OR  **behav***  OR  **"acceptance commitment"**  OR  **psychodynamic**  OR  **psychoanaly***  OR  **psychologic***  OR  **relax*** )  W/2  ( **therap***  OR  **treatment***  OR  **intervention*** )  OR  **psychotherap***  OR  **psychoeducat***  OR  **feedback***  OR  **mindfulness** ) )

AND

( ( TITLE-ABS-KEY ( **"sickness absence"**  OR  **"work resumption"**  OR  **"medical leave"**  OR  **"sick leave"**  OR  **absenteeism** ) )  OR  ( TITLE-ABS-KEY ( **return***  W/2  **work** ) )  OR  ( TITLE-ABS-KEY ( ( **vocation***  OR  **employment**  OR  **work** )  W/2  **rehabilit*** ) ) )

AND

( LIMIT-TO ( PUBYEAR ,  **2015** )  OR  LIMIT-TO ( PUBYEAR ,  **2014** )  OR  LIMIT-TO ( PUBYEAR ,  **2013** )  OR  LIMIT-TO ( PUBYEAR ,  **2012** )  OR  LIMIT-TO ( PUBYEAR ,  **2011** )  OR  LIMIT-TO ( PUBYEAR ,  **2010** )  OR  LIMIT-TO ( PUBYEAR ,  **2009** )  OR  LIMIT-TO ( PUBYEAR ,  **2008** )  OR  LIMIT-TO ( PUBYEAR ,  **2007** )  OR  LIMIT-TO ( PUBYEAR ,  **2006** )  OR  LIMIT-TO ( PUBYEAR ,  **2005** )  OR  LIMIT-TO ( PUBYEAR ,  **2004** )  OR  LIMIT-TO ( PUBYEAR ,  **2003** )  OR  LIMIT-TO ( PUBYEAR ,  **2002** )  OR  LIMIT-TO ( PUBYEAR ,  **2001** )  OR  LIMIT-TO ( PUBYEAR ,  **2000** )  OR  LIMIT-TO ( PUBYEAR ,  **1999** )  OR  LIMIT-TO ( PUBYEAR ,  **1998** )  OR  LIMIT-TO ( PUBYEAR ,  **1997** )  OR  LIMIT-TO ( PUBYEAR ,  **1996** )  OR  LIMIT-TO ( PUBYEAR ,  **1995** )  OR  LIMIT-TO ( PUBYEAR ,  **1994** )  OR  LIMIT-TO ( PUBYEAR ,  **1993** )  OR  LIMIT-TO ( PUBYEAR ,  **1992** )  OR  LIMIT-TO ( PUBYEAR ,  **1991** )  OR  LIMIT-TO ( PUBYEAR ,  **1990** )  OR  LIMIT-TO ( PUBYEAR ,  **1989** )  OR  LIMIT-TO ( PUBYEAR ,  **1988** )  OR  LIMIT-TO ( PUBYEAR ,  **1987** )  OR  LIMIT-TO ( PUBYEAR ,  **1986** )  OR  LIMIT-TO ( PUBYEAR ,  **1985** )  OR  LIMIT-TO ( PUBYEAR ,  **1984** )  OR  LIMIT-TO ( PUBYEAR ,  **1983** )  OR  LIMIT-TO ( PUBYEAR ,  **1982** )  OR  LIMIT-TO ( PUBYEAR ,  **1981** )  OR  LIMIT-TO ( PUBYEAR ,  **1980** ) )  AND  ( LIMIT-TO ( LANGUAGE ,  **"English"** )  OR  LIMIT-TO ( LANGUAGE ,  **"Norwegian"** )  OR  LIMIT-TO ( LANGUAGE ,  **"Swedish"** )  OR  LIMIT-TO ( LANGUAGE ,  **"Danish"** ) )
